# Simulation of Field Assisted Sintering of Silicon Germanium Alloys

**DOI:** 10.3390/ma12040570

**Published:** 2019-02-14

**Authors:** Anastasiia Tukmakova, Anna Novotelnova, Kseniia Samusevich, Andrey Usenko, Dmitriy Moskovskikh, Alexandr Smirnov, Ekaterina Mirofyanchenko, Toshiyuki Takagi, Hiroyuki Miki, Vladimir Khovaylo

**Affiliations:** 1 Faculty of Cryogenic Engineering, ITMO University, St. Petersburg 197101, Russia; novotelnoval@yandex.ru (A.N.); k.l.samusevich@gmail.com (K.S.); 2 Department of Functional Nanosystems and High Temperature Materials, National University of Science and Technology “MISiS”, Moscow 119049, Russia; usenko@misis.ru (A.U.); mos@misis.ru (D.M.); khovaylo@misis.ru (V.K.); smirnoff.alexandr@gmail.com (A.S.); 3 Institute of Problems of Chemical Physics RAS, Chernogolovka 142432, Russia; 4 Inenergy LLC, Moscow 115201, Russia; 5 Pushkov Institute of Terrestrial Magnetism, Ionosphere and Radiowave Propagation (IZMIRAN), Moscow 108840, Russia; 6 JSC Scientific and Production Association “Orion”, Moscow 111538, Russia; mirofianchenko@gmail.com; 7 Institute of Fluid Sciences, Tohoku University, Sendai 980-8577, Japan; takagi@ifs.tohoku.ac.jp; 8 Frontier Research Institute for Interdisciplinary Science, Tohoku University, Sendai 980-8578, Japan; miki@fris.tohoku.ac.jp; 9 National Research South Ural State University, Chelyabinsk 454080, Russia

**Keywords:** thermoelectric materials, spark plasma sintering, mechanical alloying, silicon germanium, nanocomposite, nanostructured, FEM, modeling, simulation, field assisted sintering

## Abstract

We report a numerical study of the field assisted sintering of silicon germanium alloys by a finite element method, which takes into account contact resistances, thermal expansion and the thermoelectric effect. The distribution of electrical and thermal fields was analyzed numerically, based on the experimental data collected from spark plasma sintering (SPS) apparatus. The thermoelectric properties of Si-Ge used within the simulation were considered as the function of density and the sintering temperature. Quantitative estimation of the temperature distribution during the sintering pointed to a significant, up to 60 °C, temperature difference within the specimen volume for the case of the sintering temperature at 1150 °C.

## 1. Introduction

Silicon germanium alloys have been used as a reliable material for thermoelectric generators operating in the high-temperature range. Nowadays Si-Ge is one of the most promising materials for waste heat recovery applications in industry [1]. Recent works related to Si-Ge nanostructured materials have reported significant enhancement in their thermoelectric properties as compared to the known bulk values [2,3,4,5,6,7]. Validation of the effectiveness of nanostructuring to Si-Ge has been simultaneously proved by experimental [8,9] and theoretical modeling approaches [10]. The nanostructuring has also been demonstrated to enhance thermoelectric properties of many other thermoelectrics, such as Bi_2_Te_3_, PbTe, etc. [11,12,13]. The main factor responsible for the enhancement of figure of merit (ZT) in these materials is suppression of lattice thermal conductivity due to the increased phonon scattering, as well as electron tunneling and energy filtering effects on grain boundaries [7,14,15,16,17,18,19,20,21]. 

To obtain nanostructured bulk thermoelectrics, the spark plasma sintering (SPS) technique has been frequently utilized. This is a state-of-the-art method, in which impulses of direct or alternative current and uniaxial pressure are utilized for compacting nanopowders. It is postulated that during sintering, local electric arcs arise between particles and local temporally limited areas of high temperature and pressure take place [22,23]. The sintering temperature T_s_ and the heating rate are crucial parameters, that have an impact on material grain size and densification rate.

Usually, a thermocouple or pyrometer is used for temperature control during SPS. However, this provides only a rough indication for the distribution and evolution of temperature in the specimen volume. Additional calibration or numerical study is required in order to obtain more detailed information about these processes [24]. In real SPS systems, a temperature field distribution within specimen is inhomogeneous. Significant temperature gradients can have an impact on the specimen homogeneity and, therefore, on its properties. Moreover, at higher temperature rates, temperature gradients (especially in the radial direction) can be very large due to the radiation that becomes more intensive on the die surface [24]. Temperature gradients can be decreased by the die geometry modification. For example, in Reference [25] the die geometry was changed and additional isolating elements were added to the construction in order to reduce temperature gradients in the alumina specimen.

In the case of thermoelectric materials sintering, a Peltier effect occurs and creates an additional temperature gradient that seems to be higher in the vertical direction than in the radial one. There are just a few studies, which take into account thermoelectric effects occurring in the contact area between a sample and the plungers (or a graphite die). For instance, the impact of thermoelectric effects has been experimentally and numerically studied in Reference [26] for the case of MgSi_2_ and MnSi_1.4_ samples in order to obtain a more realistic picture of the temperature field distribution. In order to trace the sintering temperature difference ΔTs in the samples, two thermocouples were placed onto the sample-plungers interfaces, and a small sector of the die was cut off. For the sintering temperatures of about 1000 °C, a vertical temperature gradient of 55 and 60 °C was found to be formed in MgSi_2_ and MnSi_1.4_ samples, respectively. The influence of the temperature gradient on thermoelectric properties of Bi_0.5_Sb_1.5_Te_3_ has been reported in Reference [27].

Numerical methods can provide an effective tool for studying the evolution of sample temperature in the course of sintering. A computer simulation based on the finite elements method (FEM) is convenient and the most commonly used method for SPS simulation [25,26,28,29,30,31,32]. Data on current, pressure and/or displacement are taken from the SPS tracking system. However, information on mechanical stresses, electrical and thermal contact resistance is usually not available or cannot be measured with sufficient accuracy. Among these, the contact resistances are of the great importance for SPS calculations. For example, based on the model that considered contact resistances, the need for calibration-based correction of the specimen temperature has been demonstrated in Reference [24]. A 2D thin layer with experimentally measured electrical and thermal resistances is usually used to simulate contact resistances. However, such an approach is very specific and requires experimental measurements for each specimen composition, sintering conditions, geometry, etc.

In the present study, a 2D axisymmetric model of Si-Ge alloys sintering was used. The model described the electrical, thermal and mechanical aspects of SPS, considering thermal expansion, thermoelectric effect and contact resistances. The method of contact resistances calculation used in this paper implies calculation of the contact pressure and takes into account the roughness of the surfaces being in the contact.

## 2. Materials and Methods

Powders (Si, Ge and B) of at least 99.99% purity were used for fabricating Si_80_Ge_20_ doped with 2% (at.) of boron. Powders were sintered with a respect of parameters optimized in previous work [4]. The samples were compressed at room temperature for two minutes, then the pressure was increased and reached the peak of 60 MPa. The samples were gradually heated to 100 °C and then the temperature was raised up to 1150 °C, with a heating rate of 15 °C/s. The soaking time was 5 min, then the pressure was reduced to 10 MPa, and the samples were slowly cooled to the room temperature. The sintering was performed in vacuum. During the consolidation cycle, the experimental parameters of temperature, applied pressure, current, voltage, and displacement were recorded continuously.

For the model calculations, several samples with the different soaking temperatures in the range of 500 °C to 1150 °C were synthesized to investigate thermal and electrical fields during the sintering process. The density was measured using the conventional Archimedes principles. Thermal diffusivity measurements were carried out using a laser flash analysis system (Netzsch LFA 457). The heat capacity was determined from differential scanning calorimetry (DSC) measurements performed by a Netzsch DSC 204 F1. The thermal conductivity κ was calculated from the results of thermal diffusivity, heat capacity and density of the samples. The electrical conductivity σ_el_ was measured on bars 1 mm × 3 mm × 12 mm using a homemade transport measuring system (Cryotel Ltd., Moscow, Russia). The accuracy of these measurements were checked against a silver sample of 99.99% purity. The values of the Seebeck coefficient were taken from Reference [4].

In the current study, we experimentally determined electrical and thermal conductivities of the sintered materials with respect to their T_s_. A line of individual samples has been sintered for each sintering temperature. Values of electrical (σ_el_) and thermal (κ) conductivities were obtained as follows. After the sintering, the disc samples were taken from the SPS setup and corresponding measurements (either thermal diffusivity or electrical conductivity) were performed in a wide temperature interval. The magnitude of σ_el_ or κ at T_s_ was taken as that one obtained from the measurement data at the corresponding temperature.

**Figure 1:**
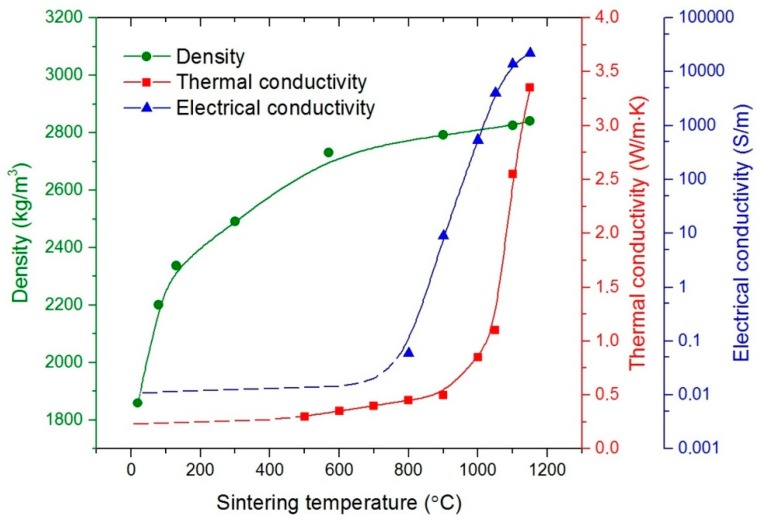
Dependence of electrical conductivity σ_el_, thermal conductivity κ and density ρ on sintering temperature of Si_80_Ge_20_ samples.

Dependences of sample density, electrical and thermal conductivities as the functions of sintering temperature are presented in Figure 1. At the initial sintering stages, the samples had high porosity and low mechanical properties. Due to this fact it was impossible to measure the values of thermal conductivity up to the *T_s_* = 500 °C and electrical conductivity up the *T_s_* = 800 °C. At temperatures lower than these values, we used approximation by the nearest function. A drastic increase of σ_el_ and κ seen for the samples sintered at *T_s_* ≥ 800 °C was conditioned by the fact that these samples had rather large relative densities, which approach to the theoretical value for *T_s_* ≥ 1100 °C [3,4,5,6,7,8,9]. 

The mechanical properties of bulk SiGe were taken from [33] and were adjusted in accordance with the sample porosity change. The Poisson ratio and Young modulus must be taken as density-dependent variables [34,35,36]. The dependences of Poisson’s ratio ν and Young’s modulus *E* on the relative density ρ*_r_* are the following [37]:

*E* = *E_s_*(*T*)ρ*_r_*^3.2^, (1)

*v* = *0.5* ρ*_r_*^2^, (2)

(3) $$\rho_{r}=\frac{V_{d}}{V}$$

where $E_{s}\left(T\right)$ is a temperature-dependent Young’s modulus of a fully dense material, *V_d_* is a volume of dense material without pores, and *V* is the material volume.

The sample was assumed to be an elastic medium, behaving in accordance with Hooke’s law.

## 3. Modeling

### 3.1. Geometry

**Figure 2:**
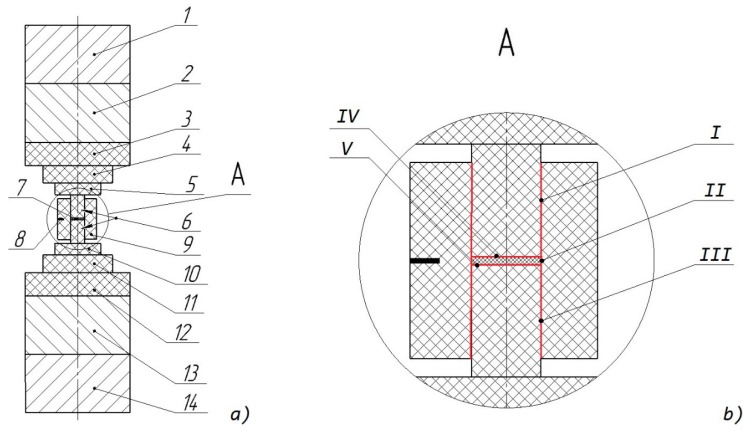
Front view of the spark plasma sintering (SPS) setup (**a**), an enlarged view of the specimen and the mold (**b**); positions I–V are contact interfaces.

The modelling was implemented in the Comsol multiphysics software. The geometry of the SPS setup used within the modelling is presented in Figure 2. Positions 1 and 14 correspond to steel electrodes; 2 and 13 to copper inserts; 3–5 and 10–12 to graphite inserts; 6—plungers; 7—sample; 8—thermocouple aperture TC; 9—graphite die. The setup, except the die and the sample, consisted of domains that were combined together using an option “form union” and no contact resistances were considered in the interfaces between the setup domains. The interfaces between setup elements and the sample, and between the die and the plungers were built as the contact pairs using an option “form assembly”. Five contact pairs were built: Two contacts between upper and lower punches and a die (positions I and III, Figure 2b), the contact between the sample and the die (position II, Figure 2b), two contacts between plungers and the sample (positions IV and V, Figure 2b).

### 3.2. Mathematical Description

#### 3.2.1. Electrical and Thermal Processes

The current density *j* and heat flux density *q* are determined as:

***j*** = −σ_el_ (∇*V*+*S*∇*T*), (4)

***q***= κ∇*T* + *ST**j***, (5)

where σ_el_ is electrical conductivity, *V* is the voltage, *S* is the Seebeck coefficient, *T* is the absolute temperature, κ is the coefficient of thermal conductivity.

The charge conservation law:

div ***j*** = 0. (6)

The heat balance equation:

(7) $$c_{p}\;\rho \frac{\partial T}{\partial t}+\mathrm{div}\;q=Q_{j}+Q_{h},$$

where *c*_p_ is the heat capacity, ρ is the density, t is the time, *Q*_j_ is the Joule heat (*Q*_j_ = j∇V), *Q*_h_ is the dissipated heat.

**Figure 3:**
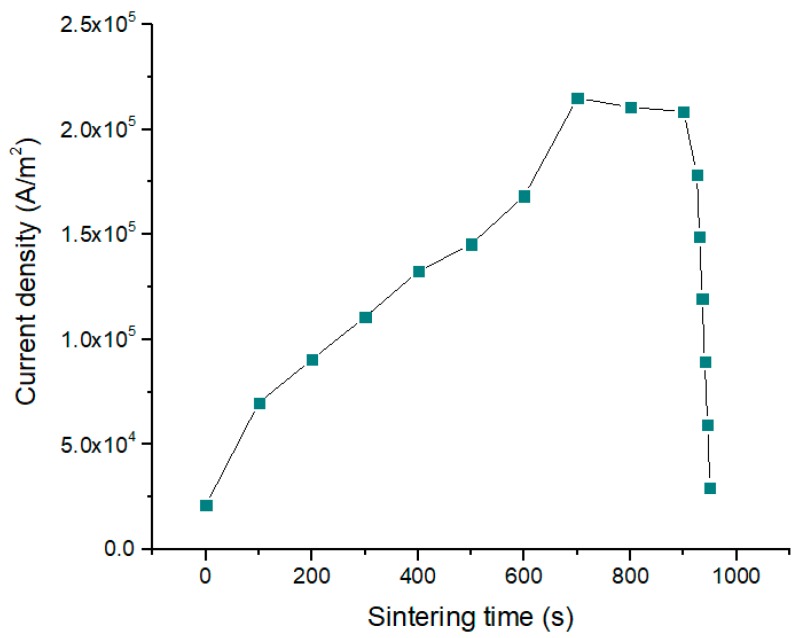
The dependence of current density on time that was used in the model with thermoelectric effect and contact resistance.

The dependence of current density on time is presented in Figure 3. 

#### 3.2.2. Mechanical Processes

The relation between stresses tensor σ_mech_ and an applied force *F* has the following form:

∇·σ_mech_ + *F* = 0. (8)

The total engineering strain tensor ε is written in terms of the displacement gradient ***u***:

ε = 0.5·(∇***u*** + ∇***u**^T^*). (9)

Hooke’s law relates the stress tensor s to the strain tensor and temperature:

*s* = *s*_0_ + C:(ε − ε_0_ − ε_th_), (10)

where *s*_0_ is the initial stress, C is the 4th order stiffness tensor or elastic moduli, “:” stands for the double-dot tensor product (or double contraction), ε_0_ is the initial strain, ε_th_ is the thermal strain, ε_th_ = α (*T* − *T*_0_)—thermal strain, where α is the coefficient of thermal expansion, *T*_0_ is an initial temperature.

#### 3.2.3. Electrical and Thermal Contacts

Almost in all papers, which consider electrical and thermal contacts, a 2D layer with specified values of thermal and electrical conductivities is used. Usually, layer properties correspond to the graphite paper/foil (e.g., Papyex foil). However, Si-Ge alloys are usually sintered without foil. 

Another way to determine the resistances is experimental measurement [38,39,40]. According to Reference [30], contact resistances in the horizontal planes between setup elements may be neglected if pressure values are higher than 50 MPa. In our case the impact of horizontal contacts were taken into consideration because the material used in the simulation was different, as well as the sintering conditions. The vertical contact resistances have been reported to be larger than horizontal ones [24].

In our model, mechanical contacts were created due to the assembly connection. Contact roughness was defined by asperities average high σ_asp_ and slope m_asp_. The m_asp_ was assumed to have the default value 0.4. It was admitted that σ_asp_ equaled the sum between the half grain size of the materials being in the contact. For the graphite used in the experiment, a half grain size equaled 22.5 μm [41]. Hence, for “graphite–graphite” contacts (interfaces I and III, Figure 2b), the average asperities height was equal to 45 μm. According to a microscopic analysis [3] the average grain size of nanostructured Si-Ge did not exceed 5 μm. A half grain size of Si-Ge was assumed to be 2.5 μm. Hence, the average asperities height for the “Si-Ge–graphite” contacts were equal to 25 μm within the calculations (interfaces II, IV, V, Figure 2b). The value of microhardness was taken for a less harder material (i.e., graphite). 

The function of contact pressure *p*_c_ vs time were calculated for the contacts. These functions were used for the contact resistances evaluation. The model of electrical and thermal contact conductance was described in [42] and presented in this work in Equations (11)–(18).

Normal current density ***j*** through the contact interface is determined as:

***n***∙***j*_1_** = −*h*_e_(*V*_1_ − *V*_2_), (11)

***n***∙***j*_2_** = −*h*_e_(*V*_2_ − *V*_1_), (12)

where *V*_1_ is voltage on the source boundary, *V*_2_ is voltage on the destination boundary.

Electrical conductance h_e_ of contact interface is:

(13) $$h_{e}=1.25\sigma_{\mathrm{el}.\mathrm{cont}.}\;\frac{m_{asp}}{\sigma_{asp}}{\left(\frac{p_{c}}{H_{c}}\right)}^{0.95},$$

where σ_el.cont_ is the harmonic mean of contact interface electrical conductivity, *H*_c_ is the hardness of the less hard material being in the contact.

Heat flux density ***q*** through the contact interface is calculated as:

−***n***∙***q*_1_** = −*h*_t_(*T*_1_ − *T*_2_), (14)

−***n***∙***q*_2_** = −*h*_t_(*T*_2_ − *T*_1_), (15)

where *T*_1_ and *T*_2_ are the temperatures of the source and destination boundaries, correspondently.

Thermal conductance *h*_t_ of contact interface is determined as:

*h*_t_ = *h*_c_ + *h*_g_ + *h*_r_, (16)

where *h_g_* and *h_r_* are the thermal conductance of the gap and the radiative conductance, respectively; *h_g_* was assumed to be zero, and:

(17) $$h_{r}=\frac{\sigma_{SB}\varepsilon_{1}\varepsilon_{2}}{\varepsilon_{1}+\varepsilon_{2}-\varepsilon_{1}\varepsilon_{2}}\left(T_{1}^{3}+T_{1}^{2}T_{2}+T_{1}T_{2}^{2}+T_{2}^{3}\right)$$

(18) $$h_{c}=1.25\;\kappa_{\mathrm{cont}}\;\frac{m_{asp}}{\sigma_{asp}}{\left(\frac{p_{c}}{H_{c}}\right)}^{0.95}$$

where σ_SB_ = 5.670 × 10^−8^ W∙m^−2^∙К^−4^ is the Stefan–Boltzmann constant, ε_1_ and ε_2_ are the emissivity factors of the source and destination boundaries being in the contact, κ_cont_ is the harmonic mean of the contact interface thermal conductivity.

#### 3.2.4. Electric Boundary Conditions

SPS setup was considered as a part of electric circuit. The experimental time-dependent electric current density was used as a current source; it was applied to the upper electrode 1 (Figure 2). The voltage on the lower electrode end 14 (Figure 2) was set to zero in order to close the circuit. The lateral setup surface was electrically isolated.

#### 3.2.5. Thermal Boundary Conditions

A convective heat exchange by means of water cooling of the upper and lower electrodes ends is:

−***n***(κ∇*T*) = *h*∙(*T*_ext_ − *T*_e_), (19)

where *h* is the coefficient of heat transfer, equal to 370 W/m^2^∙K; n is normal vector, *T*_ext_ = 20 °C is the water temperature, and *T*_e_ is the temperature on electrode surface.

The radiative heat transfer is given by:

−***n***(κ∇*T*) = ε_r_ σ_SB_ (*T*_amb_^4^ − *T*^4^), (20)

where *T*_amb_ = 20 °C is the ambient temperature.

#### 3.2.6. Mechanical Boundary Conditions

A mechanical pressure was applied to the lower steel electrode 1 (Figure 2). The upper punch surface was fixed in such a way that the displacement was equal to zero value. Lateral surfaces of the installation were free to expand or shrink in any direction.

#### 3.2.7. Mesh

**Figure 4:**
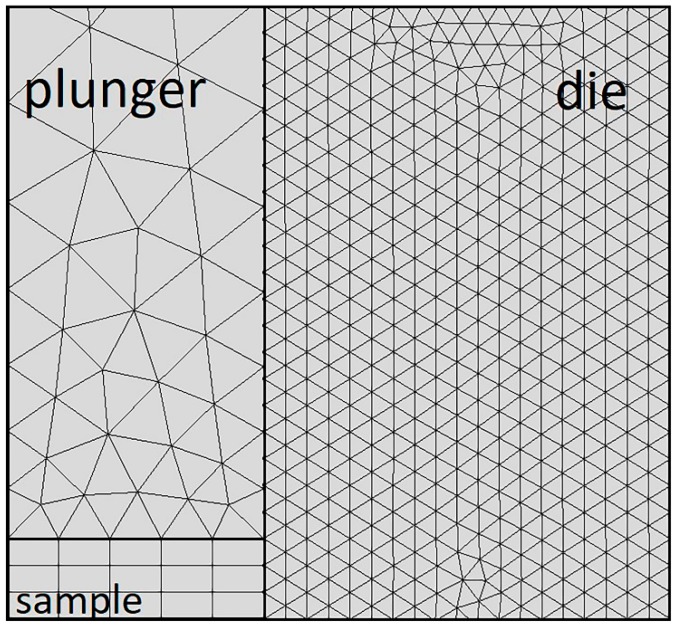
The mesh on the setup model fragment.

The modelling of contacts was accompanied by the additional difficulties in mesh building. More attention should be paid to the contact area, as no joint knots were built there. The mesh was built manually using the elements of triangular and rectangular shape. The number of mesh elements was 2240. The size of the elements on destination boundary (die, sample) was preferably to be less than that on the source boundary (plunges) (Figure 4).

## 4. Simulation Results and Discussion

### 4.1. Temperature and Current in the Sample and Setup Elements

**Figure 5:**
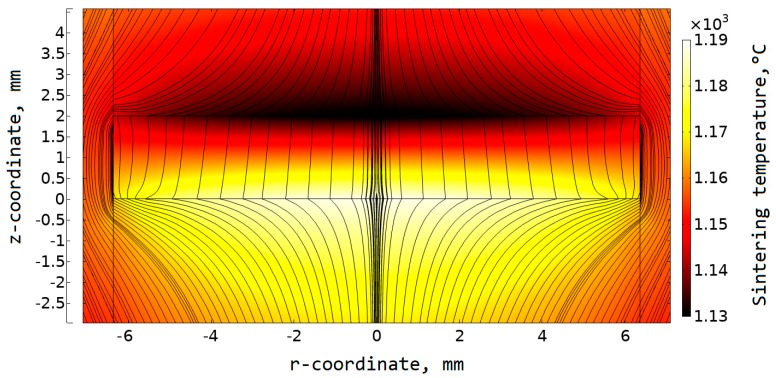
Normal current density lines in the sample and surrounding setup elements; the distribution of temperature field is presented by the 2D plot; the results are presented for *p*–type Si-Ge specimen sintered at 1150 °C.

For the convenience, a coordinate system should be set. A zero coordinate on vertical (*z*-coordinate) and horizontal (*r*-coordinate) axis (Figure 5) corresponds to the central point on the lower specimen surface.

A gradient temperature field in a sample can be seen. A Peltier heat release took place at the “specimen–lower plunger” contact (opposite to the current flow direction); a Peltier heat absorption took place on the “upper plunger–specimen” contact (along the current flow direction).

**Figure 6:**
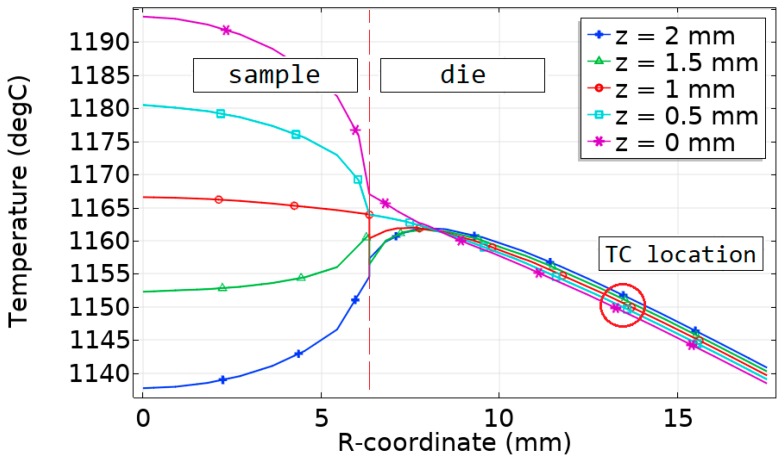
The sintering temperature in the Si-Ge sample and the mold along the radial direction for different *z*-coordinates for the sintering time *t* = 900 s. Red circle in the graph indicates the location of thermocouple aperture.

A detailed linear graph is presented in Figure 6. It shows the dependence of the sample and die temperature on the *r*-coordinate during the soaking. The maximum vertical temperature difference was as large as 61 °C, while the maximum radial temperature difference was 28 °C. 

The calculated temperature difference was compared with the results obtained experimentally on thermoelectric silicides [26]. However, the results in Reference [26] were obtained for the sample with a height of 3 mm, and with a diameter of 20 mm. Higher values of sample dimensions can result in a higher temperature difference. In our research, the Si-Ge sample height was 2 mm, with a diameter of 12.7 mm. Hence, for better comparability, we additionally simulated the sintering of Si-Ge with the sample height, which equaled 3 mm and diameter, which equaled 20 mm. The amount of Peltier heat at a point in time is directly proportional to the current and Peltier coefficient Π that can be found as Π = *S*∙*T*. Taking into account the temperature dependence of the Seebeck coefficient, its value was about 60 μV/K for MnSi_1.74_; we obtained this value using the approximation of the results reported in Reference [43]. A Seebeck coefficient of Ge-Si was about 210 μV/K at the sintering temperature. Thus, the Peltier coefficient was in the range from 290 to 316 mV for Ge-Si, and from 75 to 80 mV for MnSi_1.74_. The sintering temperature difference obtained in the Ge-Si sample with a height of 3 mm and diameter of 20 mm reached 118 degrees in comparison with 65 degrees in the MnSi_1.74_ sample of the same size. These results seemed to be in rather good correlation. Some additional parameters such as graphite properties or sintering time could have an impact on the final result.

We compared the temperature in the sample with the temperature *T*_TC_ in the point corresponding to the thermocouple aperture. The maximum difference between *T*_TC_ and *T*_s_ in the sample was 54 °C. The difference between *T*_TC_ and the average calculated temperature in the sample was 10 °C. The temperature in thermocouple aperture was closer to the colder sample surface temperature. The temperature difference was equal to 12 °C in this case.

### 4.2. Contact Resistance and Its Impact on the Temperature

**Figure 7:**
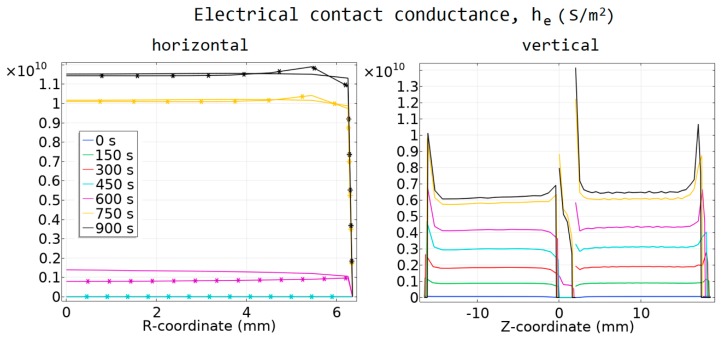
Electrical conductance of the contacts. (**a**) Contacts IV and V; asterisk markers correspond to the contact IV, solid line to contact V. (**b**) Contacts I–III.

**Figure 8:**
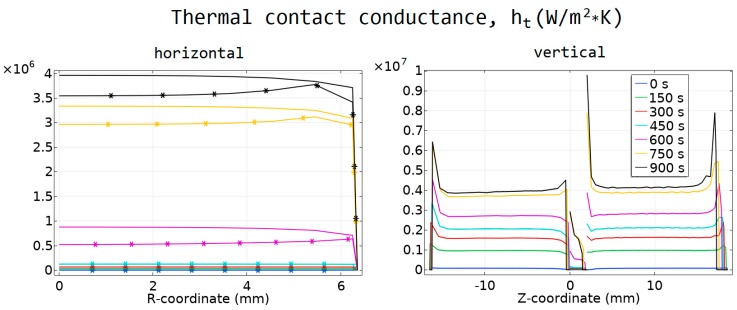
Thermal conductance of the contacts. (a) Contacts IV and V; asterisk markers correspond to the contact IV, solid line to contact V. (**b**) Contacts I–III.

In Figure 7 and Figure 8, the calculated values of the thermal and electrical contact conductance are presented. The horizontal contacts are presented as a function of the radial *r*-coordinate, and the vertical contacts as a function of the vertical *z*-coordinate.

**Figure 9:**
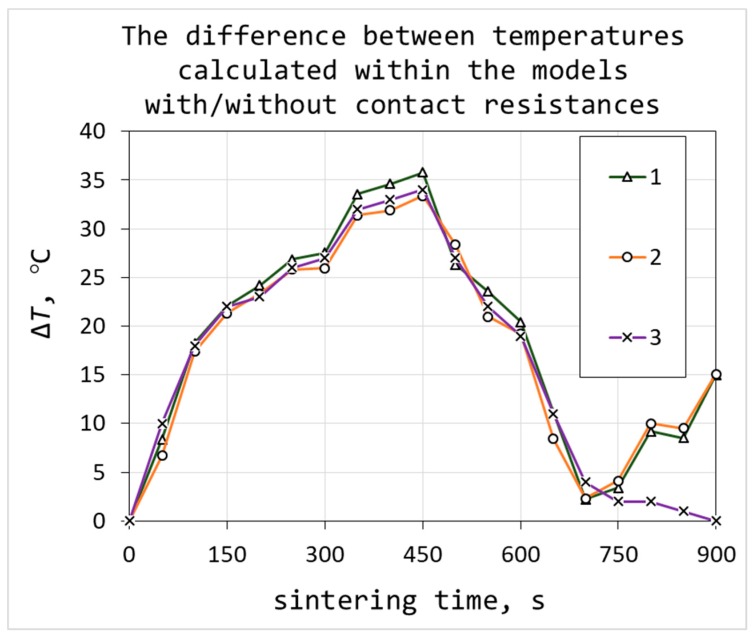
The difference between maximum sintering temperatures (1), minimum temperatures (2) and temperatures in the thermocouple (3) obtained from the models with and without contact resistances.

The comparison of the temperature values obtained from the models with and without contact resistances is presented in Figure 9. The current density used for the model with contact resistances was 0.74% higher than the current density used in the model with no contacts. The maximum and minimum sintering temperatures in the samples, as well as the calculated *T*_TC_ are higher in the model with contact resistances. This difference reached 35 degrees.

**Figure 10:**
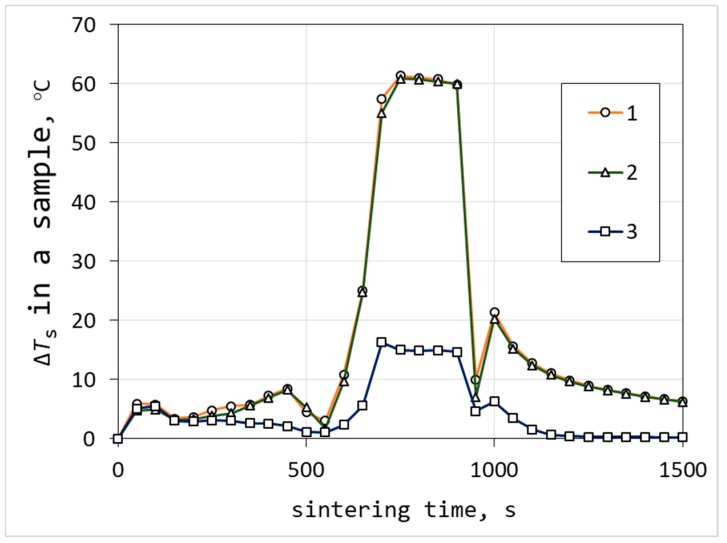
Dependence of maximum temperature difference in the sample volume on sintering time: With thermoelectric effect and contact resistances (1); with thermoelectric effect and without contact resistances (2); and without thermoelectric effect and contact resistances (3).

It is seen from Figure 10, the model with no thermoelectric effect and no contact resistances (curve 3) showed a difference of sintering temperature Δ*T*_s_ in the sample, that did not exceed 20 °C. The model with thermoelectric effect (curve 2) results in an additional 50 degrees in the Δ*T*_s_ value during the soaking time (from 700 to 900 s). At the same time, no sufficient impact of contact resistances on temperature difference was observed from the calculation.

## 5. Conclusions

The thermal conductivity and electrical conductivity of silicon germanium alloys were experimentally investigated as a function of the sintering temperature and density for the precision model calculation. FEM calculations were implemented in order to obtain a realistic picture of the temperature field in *p*-type Si-Ge samples. A convenient approach for contact resistances estimation, based on the contact interfaces roughness simulation, is presented within the model. A significant temperature difference was numerically found in the sample, due to the well-defined thermoelectric properties of Si-Ge, the impact of the Peltier effect and the contact resistances. The simulation results showed that the vertical temperature difference could reach 60 °C and the radial temperature difference could reach 30 °C during the sintering of the *p*-type Si-Ge samples.
